# Supplementary material for: Astrocyte dystrophy in ageing brain parallels impaired synaptic plasticity
Source: Aging Cell. 2021 Mar 6;20(3):e13334. doi: 10.1111/acel.13334 (PMC7963330; doi:10.1111/acel.13334)
Supplement: Supplementary file 1 — Fig S1 [file ACEL-20-e13334-s001.pdf]

Supplementary information

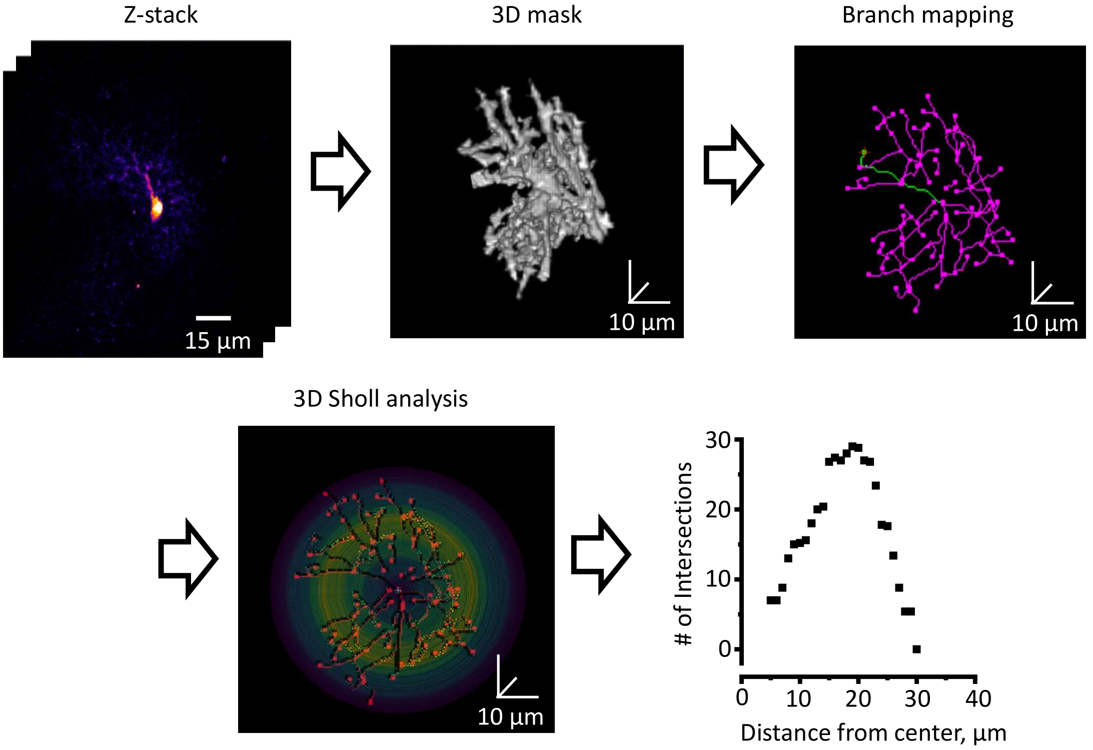

Supplementary Figure 1 Method of 3D Sholl analysis

First, a Z-stack of confocal images of astrocytes loaded with a fluorescent dye, Alexa Flo 594, through a patch pipette was obtained. Second, a three-dimensional (3D) mask of reconstructed astrocyte was obtained with a custom-written Python script. Third, the branch and branchlet mapping along the mask was performed in ImageJ (https://imagej.net/). The 3D Sholl analysis was performed with concentric spheres centered in the middle of the astrocyte soma. The number of intersections of the branches and branchlets with the spheres was obtained.
